# Supplementary material for: Learning curve and surgical outcome of robotic assisted colorectal surgery with ERAS program
Source: Sci Rep. 2022 Nov 29;12:20566. doi: 10.1038/s41598-022-24665-w (PMC9709162; doi:10.1038/s41598-022-24665-w)
Supplement: Supplementary file 2 — Supplementary Information 2. [file 41598_2022_24665_MOESM2_ESM.zip › RAL Raw data-Ñ[▒K/131/HS_ñw╝╨░O▒Kñσ.pdf]

正式摘要(電子簽章：000000231812113)		
W52 / W52 ☐ 英文標題(注意:無英文姓名-請補病人基本檔!)	☐【讀取】憑證	

臺中榮民總醫院

出院病歷摘要

轉入號： 轉出號：

[redacted]

(8)流水編號	
(9)入院日期	110/12/05 (2021)
(10)轉科日期	
(11)出院日期	110/12/11 (2021) 住院天數計 6 日

診斷

- 入院：1. Rectum cancer, Adenocarcinoma
  - s/p Ufur + short course R/T(27.5Gy/5fr. to rectal tumor and 25Gy/5 fr. to pelvic)
  2. G6PD deficiency
- 出院：1. Rectum cancer, Adenocarcinoma
  - s/p Ufur + short course R/T(27.5Gy/5fr. to rectal tumor and 25Gy/5 fr. to pelvic)
  - s/p Robotic assist LAR + loop ileostomy on 2021/12/06
  2. G6PD deficiency

主訴

- Blood tinged stool for several months

病史

- This is a 39 years old male patient with history of

  1. GPD deficiency

  According to the patient, he had blood tinged stool for a long time and the symptom progress in 2021/09. He visit CMUH where colonoscopy, CT and PET were arranged, revealed Rectal cancer. He visit several hospital for second opinion, and came to our CRS OPD on 2121/11/16. After discussion, he received Ufur + short course radiotherapy as neoadjuvant CCRT. And this time, he was admitted for surgical intervention.

體檢發現

- PS 70%

  2021/11/05 PET/CT Abnormally intense FDG uptake (SUVmax: 19.17) in the rectal region, suggesting FDG-avid malignancy (representative view in 1st image on PACS). Small LN in IMA region without FDG uptake, probably no overt metastases (2nd image on PACS).
  1101118 No side effect
  1101123 No side effect
  1101201 No side effect

  - General appearance :
    - easy looking , conscious clear , without cardiopulmonary distress
    - Vital signs自動帶入date 體溫(BT) 脈搏(P) 呼吸(R) 血壓(BP) 身高(BH) 體重(BW) BMI
      20211205 36.4℃ 71次/分 18次/分 115/70mmHg 175.3cm 68kg 22.1
    - Vital signs: B.P. 115/70 mmHg , P.R. 71 /min , R.R. 18 /min , B.T. 36.4 ℃
  - HEENT :
    - Eyes: no anemic conjunctiva, no icteric sclera, normal EOM
    - ENT: no otorrhea, no rhinorrhea, no erythematous mucosa, no injected tonsil, no deformity
  - Neck: supple, freely movable, trachea midline,no deformity, no engorged jugular vein , no palpable thyroid or lymph node enlargement
  - Chest:
    - Inspection: Grossly normal, symmetric and fully expansion, no accessory muscle used, no operation scar, no spider angiomata, no gynecomastia
    - Palpation: no palpable mass or nodule, no subcutaneous emphysema, no

- tenderness
    - Ausculation: clear breathing sound
- Heart:
    - Palpation: no thrill, no heave
    - Percussion: normal heart size
    - Ausculation: regular heart beat, normal S1, S2, no gallop, no murmur
- Extremities: No deformity, full ranged of motion without limitation, no clubbing finger or nail cyanosis, no pitting edema, no ulcer or pigmentation, no obvious skin lesion, no varicose vein, no bone, joint or muscle tenderness

CRS CONDITION

- Karnofsky performance status: (100) Normal, no complaints, no evidence of disease
- PE of Abdomen:
- CEA: 1.69 ng/ml
- Imaging Studies:
- Colonoscope report: .
- Sono , CT of abdomen and chest Films:.
- Figures:
    1. colon & rectum

手術日期及方法

- 手術日期及方法20211206 1. Robotic (XI) assisted low anterior resection
  2. Intraoperative colonoscopy : (+).
  3. High ligation (+) : At IMA root.
  4. Take down from the splenic flexure : (-)
  5. Robotic EndoGIA (45-4.3) X2 + EEA (29mm) + OpenGIA (80-3.8) X1 were applied to complete the side-to-end anastomosis
  6. Proximal cut end: > 10 cm, distal cut end: 1.5~2 cm
  7. No drainage tube was inserted
  8. ADHESION BARRIER FILM was placed below the central extended wound.
  9. Loop ileostomy (About 15~20cm from IC valve) was done over RLQ of abdomen

住院治療經過

- After admission, he received Robotic assist LAR + loop ileostomy on 2021/12/06. Under ERAS protocol, he start bland I diet on 12/07 and bland II diet on 12/08. Bland III diet on 12/10. Under relative stable condtiion, he discharged on 12/11, OPD f/u.

合併症

一般檢查

- 累積報告 :

DATE	TIME	ALB	TP	BIL.T	BIL.D	ALKP	AST	ALT	LDH	AMY	LIPASE	NH3	Ferritin	UA
1101205	1752		7.1											6.2
1101207	0209	3.4		0.6				16						

DATE	TIME	WBC	RBC	HGB	HCT	MCV	PLT	BLST	NEUT	BAND	SEG	LYM	MON	EOS	BAS
1101207	0208	7220		12.7	37.3		196		88.3			6.9	4.3	0.4	0.1

DATE	TIME	NA	K	CL	BUN	CREAT	eGFR	CA	Mg	P	CRP	ESR	PCT	D-Dimer	LACTATE
1101205	1752										0.299				
1101207	0209	142	3.9		9	0.86		8.3							

DATE	TIME	GLUCOSE	HbA1C	CHOL	TG	HDL	LDL	KETONE	CK	CKMB	TRO-T	PT-P	APTT-P	NT-pro BNP
1101205	1752					38	102							
1101205	1920			174	141									

特殊檢查

病理報告

其它

出院時情況

- 15.計劃性出院

出院指示

藥名	劑量	劑量單位	用法	頻次	數量	數量單位	現況	天數	單位
(口)Takepron OD tab 30mg	1	TAB	PO	QDAC	9	TAB	TH	9	天
Ultracet tab	1	TAB	PO	Q6HPRN	20	TAB	TH	5	天

出院計劃

- 門診日期
  - date on 2021/12/21

主治醫師 2062F 蔣鋒帆 / 住院醫師 2339C 蔡濬樟

正式摘要(電子簽章：000000231812113)

W52 / W52

doctorRank:B
